# Synoviocyte-Derived Extracellular Matrix and bFGF Speed Human Chondrocyte Proliferation While Maintaining Differentiation Potential

**DOI:** 10.3389/fbioe.2022.825005

**Published:** 2022-05-24

**Authors:** Rachel D. Truong, Megan A. Bernier, James E. Dennis, Thomas J. Kean

**Affiliations:** ^1^ College of Medicine, University of Central Florida, Orlando, FL, United States; ^2^ Department of Orthopedic Surgery, Baylor College of Medicine, Houston, TX, United States; ^3^ Biionix Cluster, Internal Medicine, College of Medicine, University of Central Florida, Orlando, FL, United States

**Keywords:** synoviocyte matrix, human chondrocyte, human chondrogenesis, chondrocyte expansion, cartilage tissue engineering, fibroblast growth factor, arthritis, osteoarthritis

## Abstract

Improving the ability of human chondrocytes to proliferate, while maintaining their differentiation potential, has presented a great challenge in cartilage tissue engineering. In this study, human chondrocytes were cultured under four unique growth conditions at physiologic oxygen tension: tissue culture plastic (TCP) only, synoviocyte matrix (SCM)–coated flasks only, SCM-coated flasks with bFGF media supplement, and TCP with bFGF media supplement. The results indicated that, compared to standard TCP, all test conditions showed significantly increased cell expansion rates and an increase in both glycosaminoglycan (GAG) and collagen content during redifferentiation culture. Specifically, the combined SCM + bFGF growth condition showed an additive effect, with an increase of approximately 36% more cells per passage (5–7 days) when compared to the SCM alone. In conclusion, the results of this study demonstrate that bFGF and SCM can be used as supplements to enhance the growth of human chondrocytes both as individual enhancers and as a combined additive.

## Introduction

Osteoarthritis (OA) is characterized as a chronic degenerative joint disease that increases in prevalence with age, afflicting the majority of patients over age 65 as displayed by radiographic evidence (Oliveria et al., 1995). Treatment modalities are aimed first at addressing symptoms through nonpharmacologic methods such as weight management, braces and then pharmacologically through pain relievers such as nonsteroidal anti-inflammatory drugs and steroid injections (Bennell et al., 2012). If both non-pharmacologic and pharmacologic methods fail, joint replacement surgery can be performed, a technique for which OA is the leading indication (Robertsson et al., 2010). A randomized controlled trial performed by Skou et al. (2015) has shown that total knee replacement followed by nonsurgical treatment proved to be more effective at reducing pain and improving function at a 12-month follow-up than nonsurgical treatment alone. However, it was also noted in the same trial that patients who were assigned total knee replacement reported a higher number of serious adverse events. It, therefore, becomes imperative to find a treatment that is just as effective as total joint replacement but reduces the risk of adverse events and overcomes the procedure’s limited lifetime of 15–20 years. This limited lifetime is especially concerning for patients aged <65 years as there is a distinct possibility that their total joint replacement could fail and require further invasive procedures before they die.

One such avenue of treatment is to use human articular chondrocytes (HACs) to repair the tissue in the diseased joint. Autologous chondrocyte implantation (ACI) and matrix-assisted autologous chondrocyte implantation (MACI) are both generally accepted techniques that have shown promise in the treatment of symptomatic chondral defects of the knee (Bartlett et al., 2005). However, human chondrocytes used in these techniques are known to de-differentiate quickly, losing their ability to form the hyaline-like cartilage tissue necessary to help treat large OA lesions (Giovannini et al., 2010). This leads to a decrease in hyaline cartilage production with time, implying that, while ACI and MACI are commonly used to delay total joint replacement, these methods may not be able to completely obviate the eventual need for total joint replacement.

Maintaining cell potency, therefore, becomes a vital goal for the purposes of tissue engineering. A synoviocyte-derived extracellular matrix has previously been shown to create an environment ideal for the proliferation of human chondrocytes without the loss of the re-differentiation potential (Kean and Dennis, 2015). Similarly, media supplemented with bFGF have been shown to cause both human mesenchymal stem cells and rabbit chondrocytes isolated from both auricular and articular sources to proliferate more rapidly than when exposed to control conditions (Solchaga et al., 2005; Mounts et al., 2012). Sieber et al. (2020) noted that, because of its avascular nature, the articular cartilage is supplied oxygen *via* diffusion from the surrounding synovial fluid, leading to oxygen tension of the tissue varying from 2–9%. Multiple groups have also previously shown that oxygen tension in the articular cartilage ranges from 2–5% (Grant and Smith, 1963; Lund-Olesen, 1970; Brighton and Heppenstall, 1971; Pattappa et al., 2019). Zhou et al. (2004) have also previously demonstrated *via* a modeling approach that the rate of oxygen consumption in the articular cartilage fell in an oxygen tension–dependent manner once the tissue oxygen tension fell below 5%. The use of oxygen tension at 5% has been shown by multiple groups, including our own, to be beneficial for chondrocyte culture (Lafont et al., 2008; Das et al., 2010; Kean and Dennis, 2015; Anderson et al., 2018; Dennis et al., 2020). Oxygen tension of 5% was, therefore, chosen because of this decreased level within the range of oxygen tensions reported as physiological.

This study examines the potential of promoting HAC growth under physiological environmental conditions (5% oxygen tension) with varying combinations of bFGF and the synoviocyte matrix (SCM). It also aims to investigate the possibility of maintaining HAC differentiation potential while undergoing growth under such conditions. The results of this study will indicate if a growth environment enhanced with the SCM and bFGF will lead to increased human chondrocyte proliferation while maintaining differentiation potential. Success in such a growth environment demonstrates the potential for the use of the SCM + bFGF in engineering cartilage lesion treatment modalities, addressing the fibrocartilage seen in ACI and MACI techniques.

## Materials and Methods

### Ethics Statement

Human chondrocytes were obtained from total joint replacement surgery discarded tissue with written informed consent under IRB-approved protocols from Case Western Reserve University and Baylor College of Medicine: IRB # IRB08-00104 and H-36374, respectively.

### Human Chondrocyte Isolation

The discarded surgical tissue of patients who had undergone total knee replacement was collected (*n = 5*). Macroscopically normal cartilage tissue was dissected from the joint under sterile conditions and minced into pieces measuring <1 mm^3^. These pieces were then sequentially digested, first with hyaluronidase (30 min, 660 U/ml in DMEM; Sigma) and then with collagenase (overnight; 583 U/ml in DMEM with 10% FBS; Worthington Biochemical). This resulted in a cell slurry which was washed through a 70-μm filter with DMEM, centrifuged (600 RCF, 5 min) and plated (6000 cells/cm^2^) into tissue culture flasks in growth media (DMEM supplemented with 10% FBS 1% pen/strep) from which the primary cell culture (p0) cell stocks were produced. Once the tissue culture flasks reached 90% confluence, they were trypsinized and cryogenically stored (95% FBS, 5% DMSO).

### Synoviocyte Matrix Production

Synoviocyte matrix–coated flasks were prepared as previously described (Kean and Dennis, 2015). Briefly, porcine synoviocytes were thawed from frozen stocks and cultured in a growth medium (DMEM-LG supplemented with 10% fetal bovine serum (FBS) and 1% penicillin/streptomycin) until 80%–90% confluent. The growth medium was then switched to DMEM-LG with FBS and ascorbate-2-phosphate (50 µM) for 5–7 days. Ascorbate-2-phosphate was included to increase ECM production, as previously carried out by Pei and He (2012), although it is understood that this can impact other aspects of the cell culture. After this period, the flask was washed with PBS and then frozen in a dry ice 100% ethanol bath. Dry ice–cooled ethanol was then used to devitalize the cell layer by pipetting into the flask; after 1 min, ethanol was removed and the flask was inverted to dry. The synoviocyte matrix was then stored at 4°C until ready for use in HAC growth.

### Chondrocyte Expansion and Aggregate Production

**FIGURE 1:**
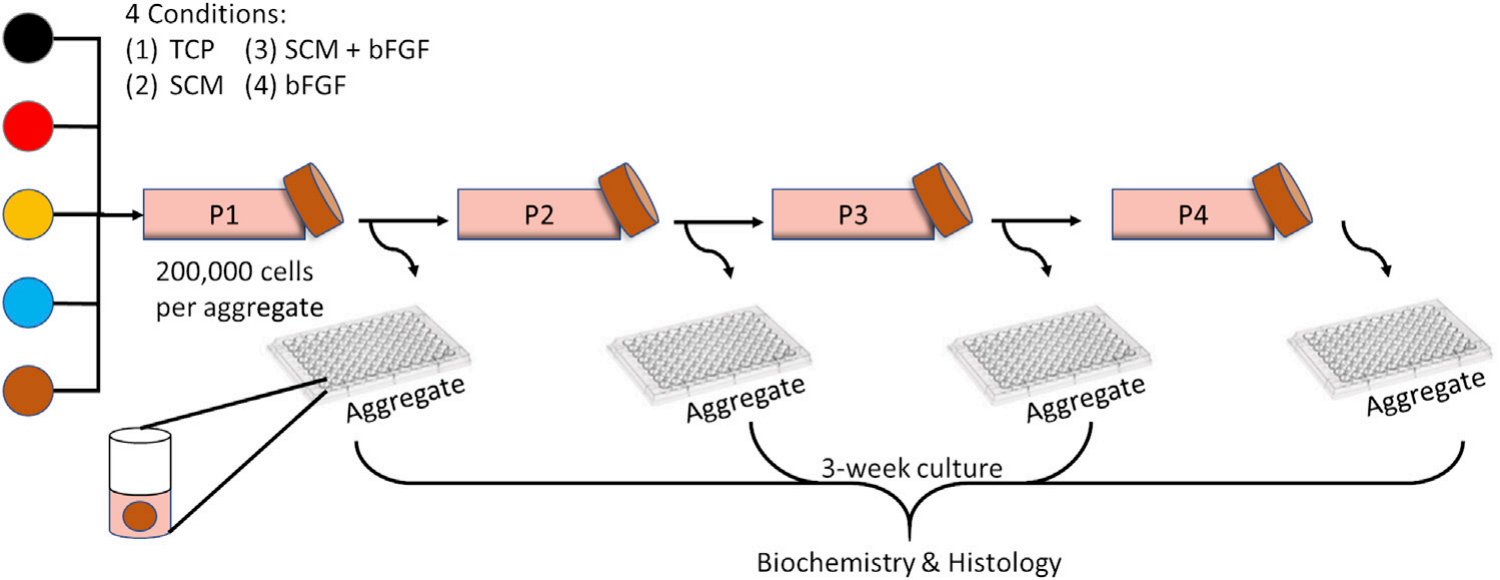
Experimental overview. Human chondrocytes from five donors (each color represents a donor) were expanded for four passages on tissue culture plastic (TCP), synoviocyte-derived extracellular matrix (SCM), on synoviocyte matrix with bFGF (SCM + bFGF), or on tissue culture plastic with bFGF (bFGF). At the end of each expansion period, the chondrocytes were trypsinized and seeded as aggregates and passaged to a new flask of the same type.

Chondrocytes were thawed from frozen stocks and seeded (6000 cells/cm^2^) into four separate flasks with unique growth conditions: 1) tissue culture plastic (TCP) only, 2) SCM-coated flasks only, 3) SCM-coated flasks with bFGF (10 ng/ml) media supplement, and 4) TCP with bFGF (10 ng/ml) media supplement (Figure 1). The bFGF concentration was chosen based on a previous study (Solchaga et al., 2005). All samples were cultured at a physiologic oxygen tension of 5% and in a growth medium of DMEM-LG with 10% FBS and 1% penicillin/streptomycin. Once the cell population in the fastest growth environment reached 85–99% confluence, all flasks were trypsinized and passaged (0.25% trypsin/EDTA; Gibco). The cells were counted with a hemocytometer, and population doublings were calculated using the formula PD = [log10 (harvested cell count)—log10 (seeded cell count)] x 3.32, and sequential passages were summed to give cumulative population doublings (Kean and Dennis, 2015). Cartilage aggregates were formed in addition to passaging a subset of these cells into new flasks of the same type (6000 cells/cm^2^). This process was repeated three more times for a total of four passages (Figure 1). Cartilage aggregates were formed as previously described (Kean and Dennis, 2015). Briefly, 200,000 cells per well in sterile nonadherent 96-well plates were condensed into aggregates by centrifugation (600 RCF, 5 min). Cell aggregates were cultured in defined chondrogenic media (DMEM-HG (Hyclone) supplemented with 1% ITS + premix (BD Biosciences), 1 % glutamax (Gibco), 1% pyruvate (Gibco), 1% nonessential amino acids (Gibco), 1% penicillin/streptomycin (Gibco), 0.5% fungizone (Gibco), dexamethasone (100 nM, Sigma), ascorbate-2-phosphate (130 μM, Wako Chemicals), and TGFβ1 (10 ng/ml) for 21 days with the medium changed every other day.

### Biochemical Assays

At the end of the 21-day culture period, cartilage aggregates were assessed for wet weight, DNA, and glycosaminoglycan and hydroxyproline content as previously described (Dennis et al., 2020). Briefly, the aggregates were digested with papain, and then the digests were split between DNA/glycosaminoglycan (GAG) assay and hydroxyproline (HDP) assays. DNA was determined from the digest using Hoechst solution (0.667 μg/ml in 0.2 M pH 8.0 phosphate buffer; 33258; Sigma Aldrich). GAG was determined using a dot blot assay and binding/extraction of safranin-O. HDP was determined from the acid-hydrolyzed digest using Ehrlich’s reagent. The collagen content was estimated from the HDP concentration by a conversion factor of 7.6 (Venn and Maroudas, 1977).

### Histology

Aggregates were formalin-fixed overnight and then transferred to 70% ethanol, dehydrated through graded ethanol and xylene to paraffin, and then paraffin-embedded. The paraffin sections (8 µm) were deparaffinized and hydrated before staining with safranin-O (Sigma Aldrich) for GAG with a Fast Green (AA16520-06, Alfa Aesar) counterstain.

Immunohistochemistry: Paraffin sections were deparaffinized and hydrated and then incubated with pronase (1 mg/ml, 10 min, room temp, Sigma). The sections were rinsed in PBS and then blocked with BSA (3%, 15 min, RT, BSA Fraction V, Omnipur). The sections were incubated overnight at 4°C with primary antibodies: type I collagen (Clone II-4CII, MP Biomedical 631703), type II collagen (DSHB II-II6B3), and type X collagen (kind gift of Gary Gibson, Henry Ford Hospital, Detroit, MI). After overnight incubation, the sections were rinsed with PBS, blocked with 3% BSA (15 min, RT), and then incubated with a secondary antibody (1 h, RT, biotinylated horse anti-mouse; Vector labs; BA2000). The slides were then rinsed with PBS and incubated with streptavidin-HRP (30 min, RT, Invitrogen SNN1004). Then, the slides were rinsed with PBS and incubated with Vector VIP Peroxidase (10 min, RT, Vector labs) before counterstaining in Fast Green and xylene mounting.

### Statistics

Each donor was expanded and multiple aggregates were formed at the end of each passage (3–8 aggregates per donor/passage). The mean value for each donor is represented by a colored symbol in figures, with the overall mean from all donors and standard deviation shown. Two-way repeated measures ANOVA was performed with matching across the passage and surface type (GraphPad Prism). Post-hoc analysis of ANOVA was corrected for multiple comparisons using Tukey’s method (GraphPad Prism).

## Results

**FIGURE 2:**
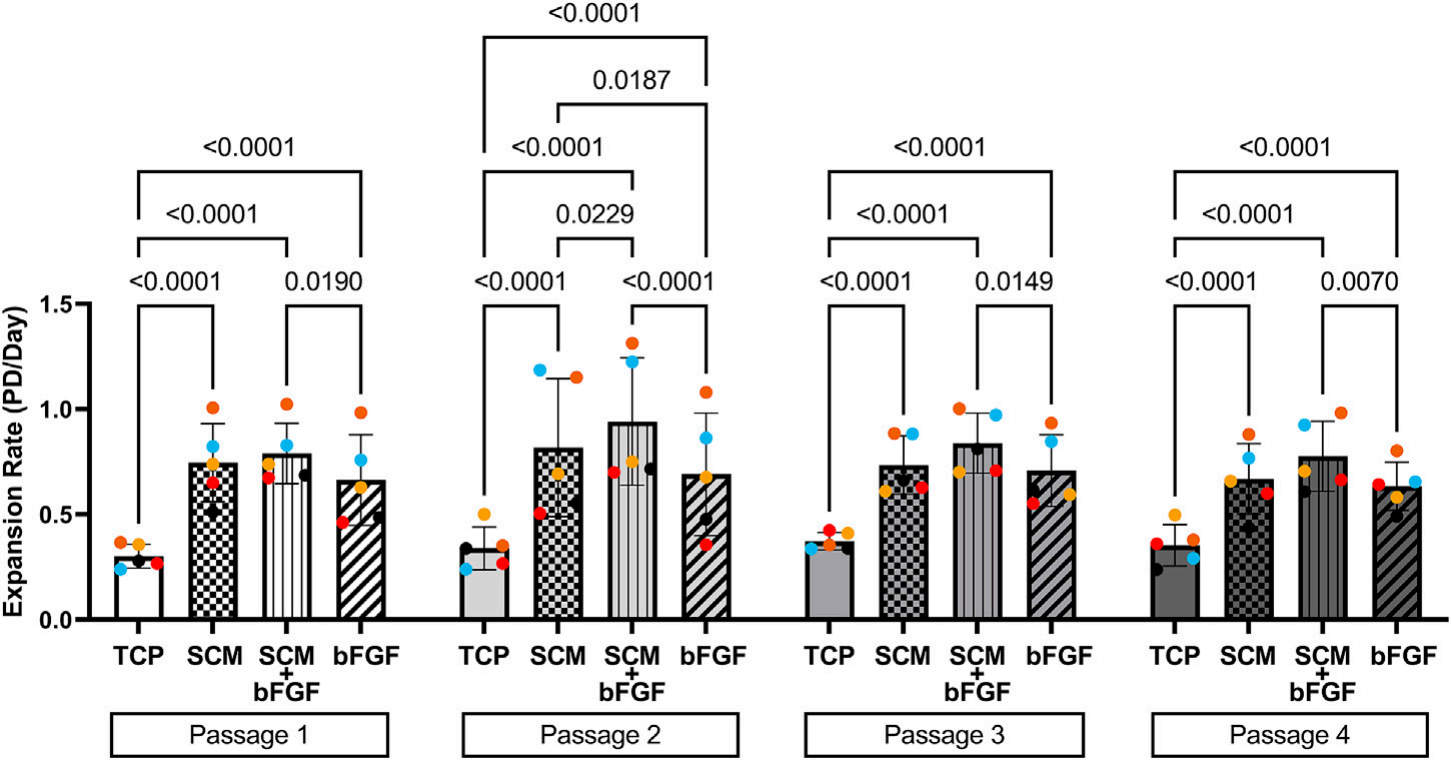
Expansion of human chondrocytes. Human chondrocytes from five donors (each color represents a donor) for four passages on tissue culture plastic (TCP), synoviocyte-derived extracellular matrix (SCM), on synoviocyte matrix with bFGF (SCM + bFGF), or on tissue culture plastic with bFGF (FGF). *p* < 0.05 are shown, two-way ANOVA.

Human articular chondrocytes from five donors were grown in each of the four culture conditions described: TCP, SCM, SCM + FGF, and FGF (Figure 1). To determine how proliferation was impacted by these culture conditions, the expansion rate was measured over four passages. The cells expanded significantly quicker in all test conditions across all four passages versus the TCP control (Figure 2). SCM + bFGF stimulated proliferation the most in all passages, surpassing that by both SCM and bFGF alone. The addition of FGF or SCM alone significantly increased the expansion rate vs. TCP at all four passages.

The cell aggregates were formed after passaging cells from each condition independently. These aggregates were tested for wet weight (Figure 3), collagen content (Figure 4), and GAG content (Figure 5). Additional replicates were also used for histological assessment (Figure 6 and Supplementary Figures S2–S13). The aggregate wet weight was similar across all conditions but showed some donor dependence with one donor being consistently higher than the mean +S.D. (Figure 3). The aggregate wet weight negatively correlated with the passage, with a trend showing decreasing wet weight (Figure 3 and Supplementary Figure S1).

**FIGURE 3:**
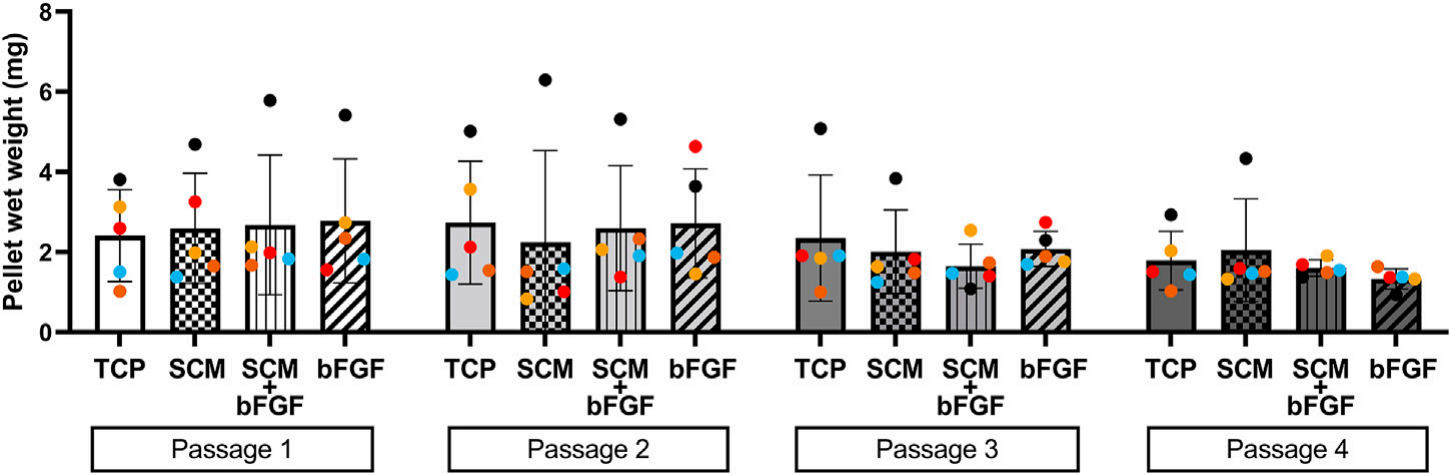
Human cartilage aggregate wet weight. Cartilage aggregates from five donors (average aggregate weight, ≥three aggregates per donor, each color represents a donor) for four passages on tissue culture plastic (TCP), synoviocyte-derived extracellular matrix (SCM), on synoviocyte matrix with bFGF (SCM + bFGF), or on tissue culture plastic with bFGF (bFGF). *p* < 0.05 are shown, two-way ANOVA (no significant differences found).

**FIGURE 4:**
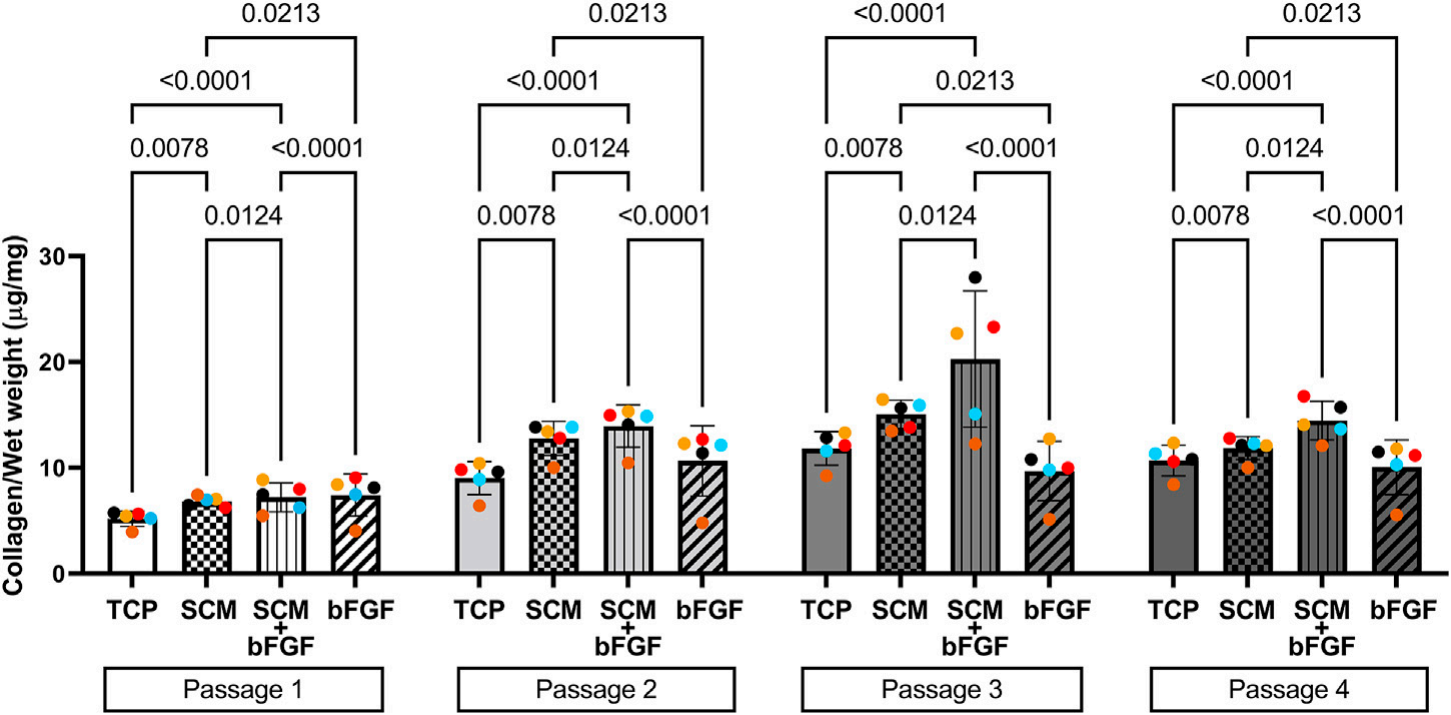
Collagen content of human cartilage aggregates. Cartilage aggregates from five donors (average aggregate collagen content per mg wet weight, ≥two aggregates per donor, each color represents a donor) for four passages on tissue culture plastic (TCP), synoviocyte-derived extracellular matrix (SCM), on synoviocyte matrix with bFGF (SCM + bFGF), or on tissue culture plastic with bFGF (bFGF). *p* < 0.05 are shown, two-way ANOVA.

**FIGURE 5:**
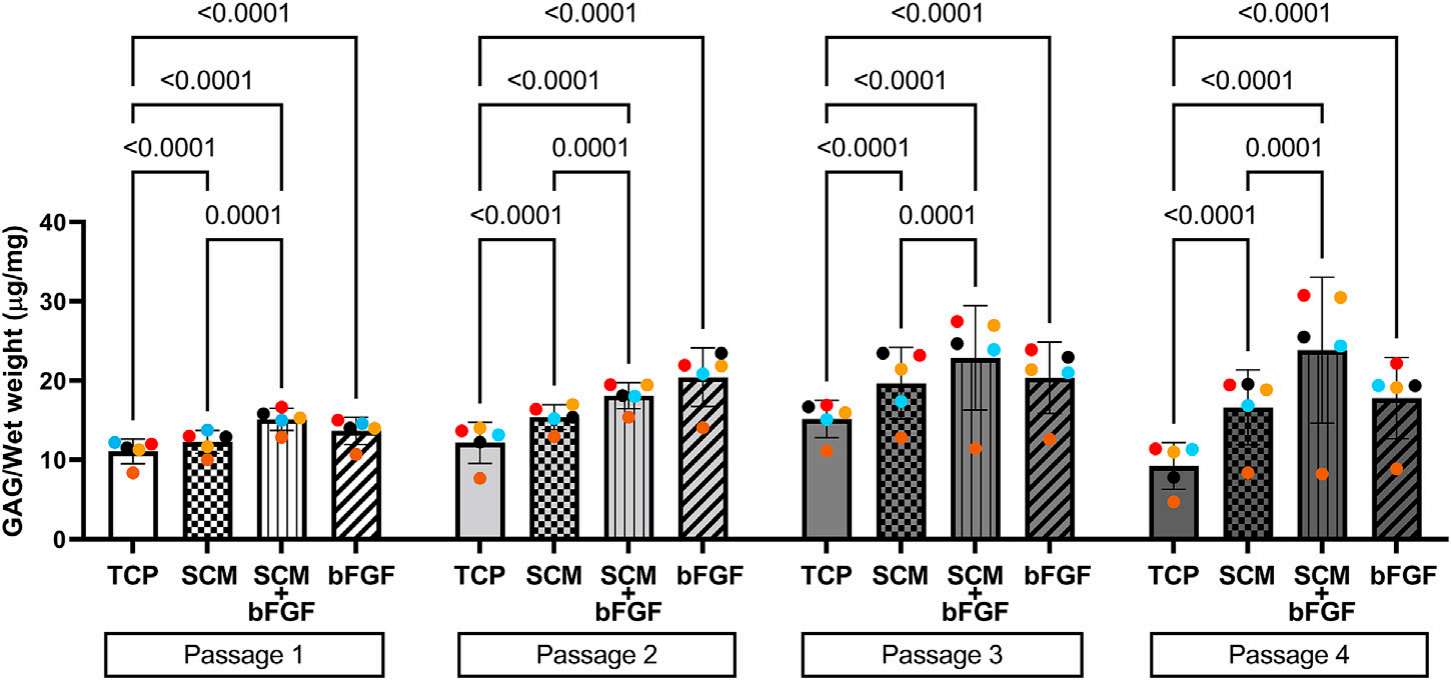
Glycosaminoglycan (GAG) content of human cartilage aggregates. Cartilage aggregates from five donors (average aggregate GAG content per mg wet weight, ≥two aggregates per donor, each color represents a donor) for four passages on tissue culture plastic (TCP), synoviocyte-derived extracellular matrix (SCM), on synoviocyte matrix with bFGF (SCM + bFGF), or on tissue culture plastic with bFGF (bFGF). *p* < 0.05 are shown, two-way ANOVA.

**FIGURE 6:**
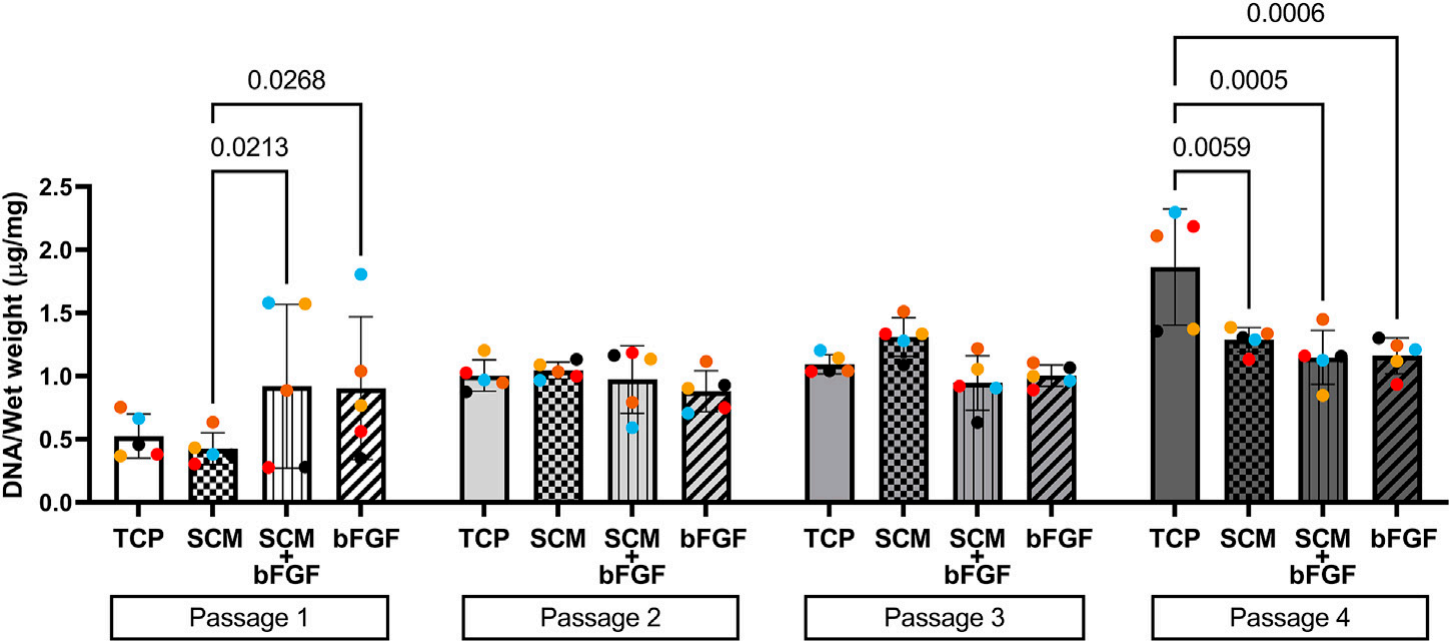
DNA content of aggregates. DNA was extracted from cartilage aggregates and normalized to wet weight (five donors, ≥2 aggregates per donor, each color represents a donor) at each of four passages on tissue culture plastic (TCP), synoviocyte-derived extracellular matrix (SCM), on synoviocyte matrix with bFGF (SCM + bFGF), or on tissue culture plastic with bFGF (bFGF). *p* < 0.05 are shown, two-way ANOVA.

To determine if the growth conditions tested impacted the collagen content of the cell aggregates, the hydroxyproline concentration was measured and used to estimate the collagen content. In passage 1, SCM + bFGF and bFGF alone had the highest levels of collagen (Figure 4). However, in the subsequent passages, the SCM and SCM + bFGF groups are the highest. SCM + bFGF has significantly higher collagen content only in passages 3 and 4 than all other groups (Figure 4). TCP alone has significantly lower collagen content in all passages except in passage 3 where no significant difference was observed other than that of bFGF (Figure 4). Overall, the collagen content tended to increase in all groups as the passage number increases.

The other critical component of the cartilage, GAG, was also quantified from cell aggregates formed at each passage. Overall, the GAG concentration increased in all groups in passages 1 through 3, with a slight decrease in passage 4 by all groups except SCM + bFGF, which remained consistent (Figure 5). TCP alone had significantly lower GAG content than all other groups in all four passages (Figure 5). In passage 1, SCM + bFGF and bFGF have a similar level of GAG, but in passage 2, FGF has a slight increase. However, in passages 3 and 4, SCM + bFGF has the highest level of GAG expression.

To assess aggregate cellularity, DNA content per milligram wet weight of aggregates was also assessed (Figure 6). In general, the DNA content increased with the passage across all conditions, signifying decreased production of the extracellular matrix per cell. There was a significant effect of the passage as a source of variability (*p* = 0.0007) and an interaction between the culture conditions and passages (*p* = 0.0002), but there was no significant effect of the culture condition on the aggregate DNA content (two-way ANOVA).

**FIGURE 7:**
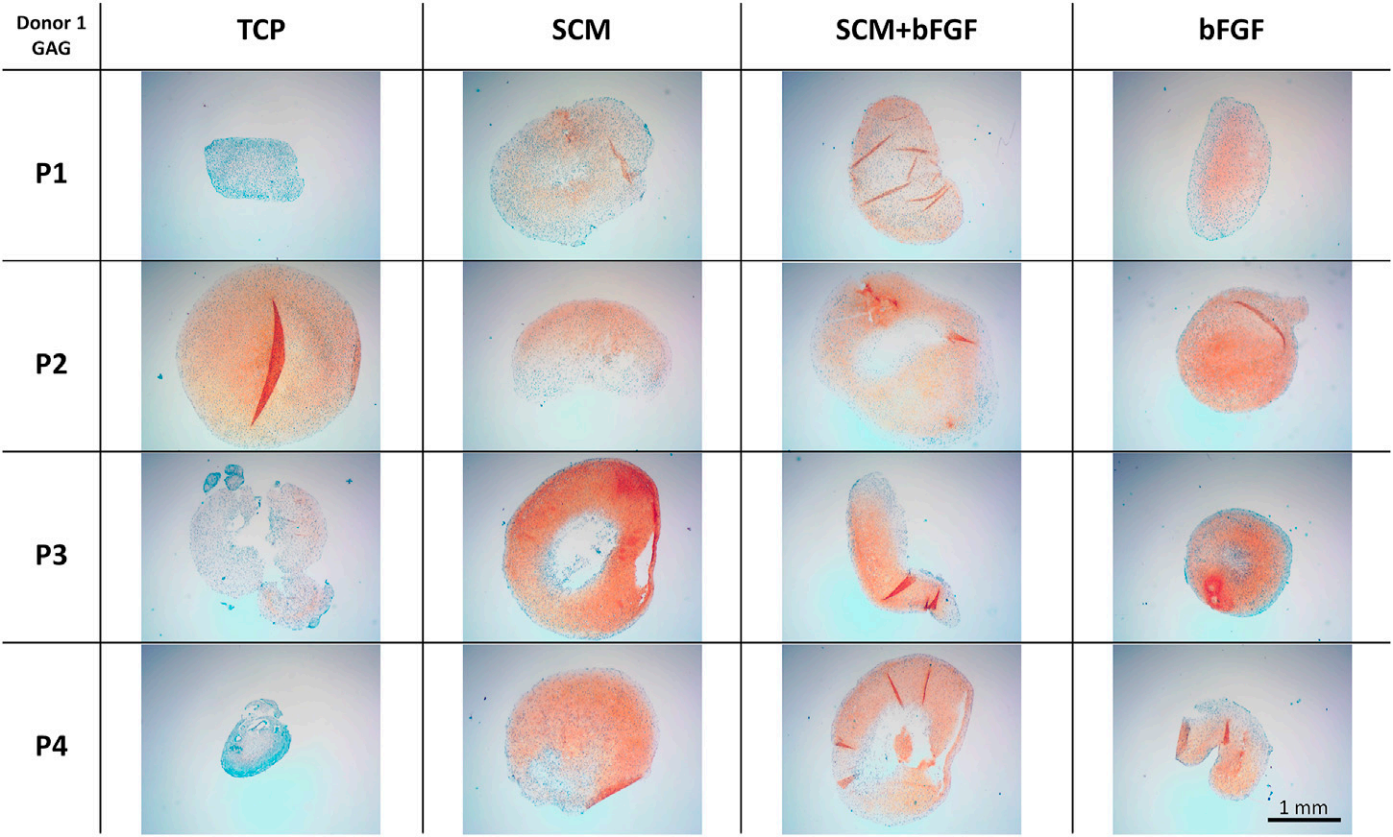
Cartilage aggregate images showing glycosaminoglycan (GAG) staining. Sections of cartilage aggregates from a single donor over the four passages on each surface are shown here stained with safranin-O/Fast Green. All at the same scale, scale bar 1 mm. See Supplementary Figures S2–S4 for other donors.

Cell aggregates were also used for the histological assessment of GAG by safranin-O/Fast Green staining. The same trends observed in the quantification of GAG content are seen in the histology staining. TCP alone has lighter staining at three of the four passages (Figure 7). The SCM alone has the darkest staining at passage 3 but is darker overall than TCP. SCM + bFGF has slightly darker staining at passage 3 versus other passages (Figure 7). The SCM, SCM + bFGF, and bFGF show slightly darker staining in passage 1 than TCP. While both the SCM and SCM + bFGF seem to have darker staining as the passages increase, TCP and bFGF seem to have lighter staining (Figure 7). There were apparent differences between donors, with donor 5 exhibiting much less GAG staining (Supplementary Figure S4). Across donors and conditions, the lag in GAG expression at P1 is most evident in TCP-derived chondrocytes but is present in all conditions (Figure 7 and Supplementary Figures S2–S4). The differences between donor, passage, and condition are less evident with donors 1–3 on the SCM, SCM + bFGF, and bFGF, respectively, with all three donors having GAG staining at passage 4 under those conditions (Figure 7 and Supplementary Figures S2–S4).

**FIGURE 8:**
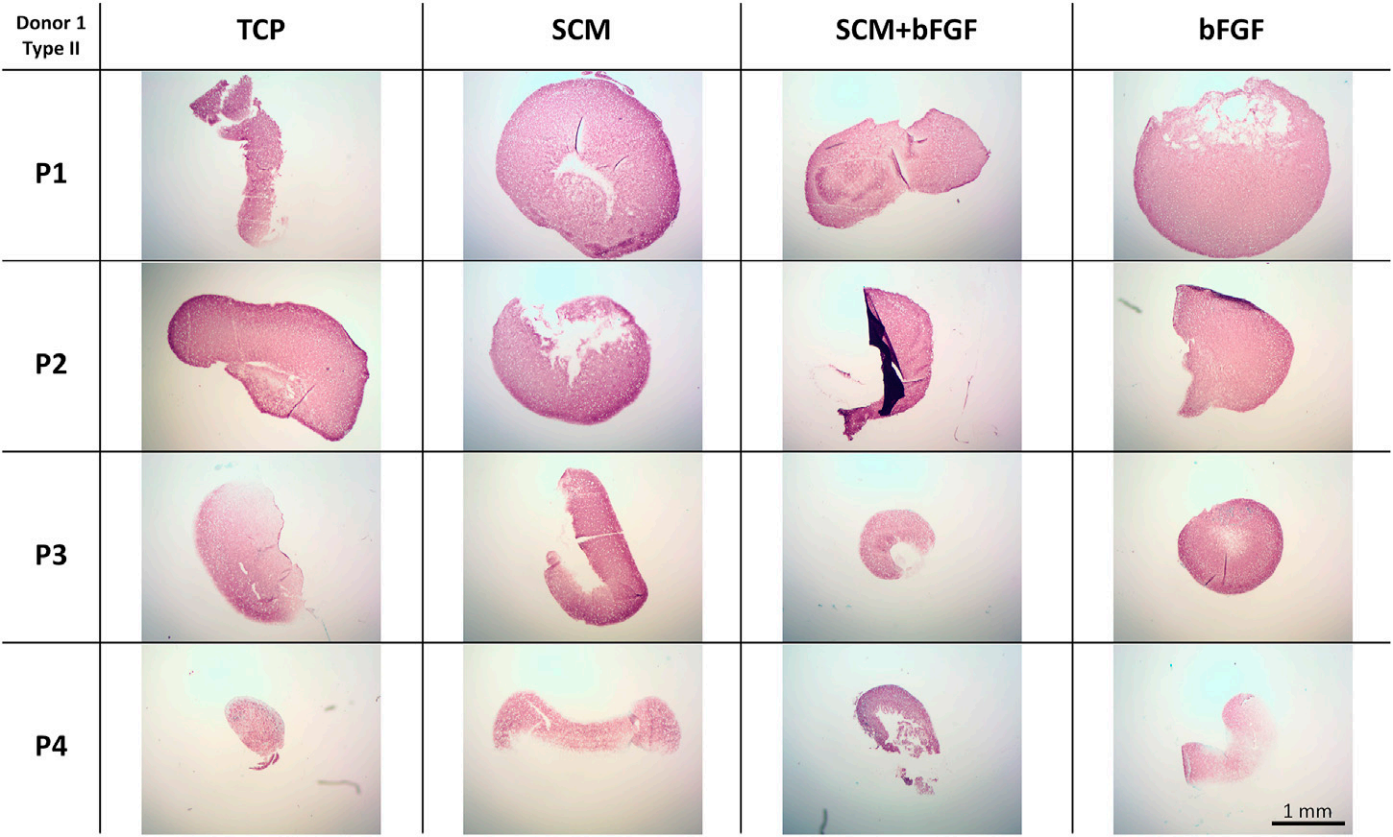
Cartilage aggregate images showing type II collagen staining. Sections of cartilage aggregates from a single donor over the four passages in each condition are shown here stained for type II collagen. All at the same scale, scale bar 1 mm. See Supplementary Figures S5–S7 for other donors.

To further investigate the extracellular matrix deposited by the chondrocytes during aggregate culture, immunohistochemistry was performed. Staining was observed for type II collagen across all conditions at all passages (Figure 8). Slightly weaker staining at passage 4 is evident in aggregates made from cells cultured with bFGF. Across the donors, donor 5 again shows relatively weak staining in all conditions (Supplementary Figure S7). The SCM and SCM + bFGF appear to have the most intense staining across all passages and donors (Figure 8 and Supplementary Figures S5–S7). A similar donor- and passage-dependent extracellular matrix expression as was seen in the GAG images is evident (Figure 8 and Supplementary Figures S5–S7).

**FIGURE 9:**
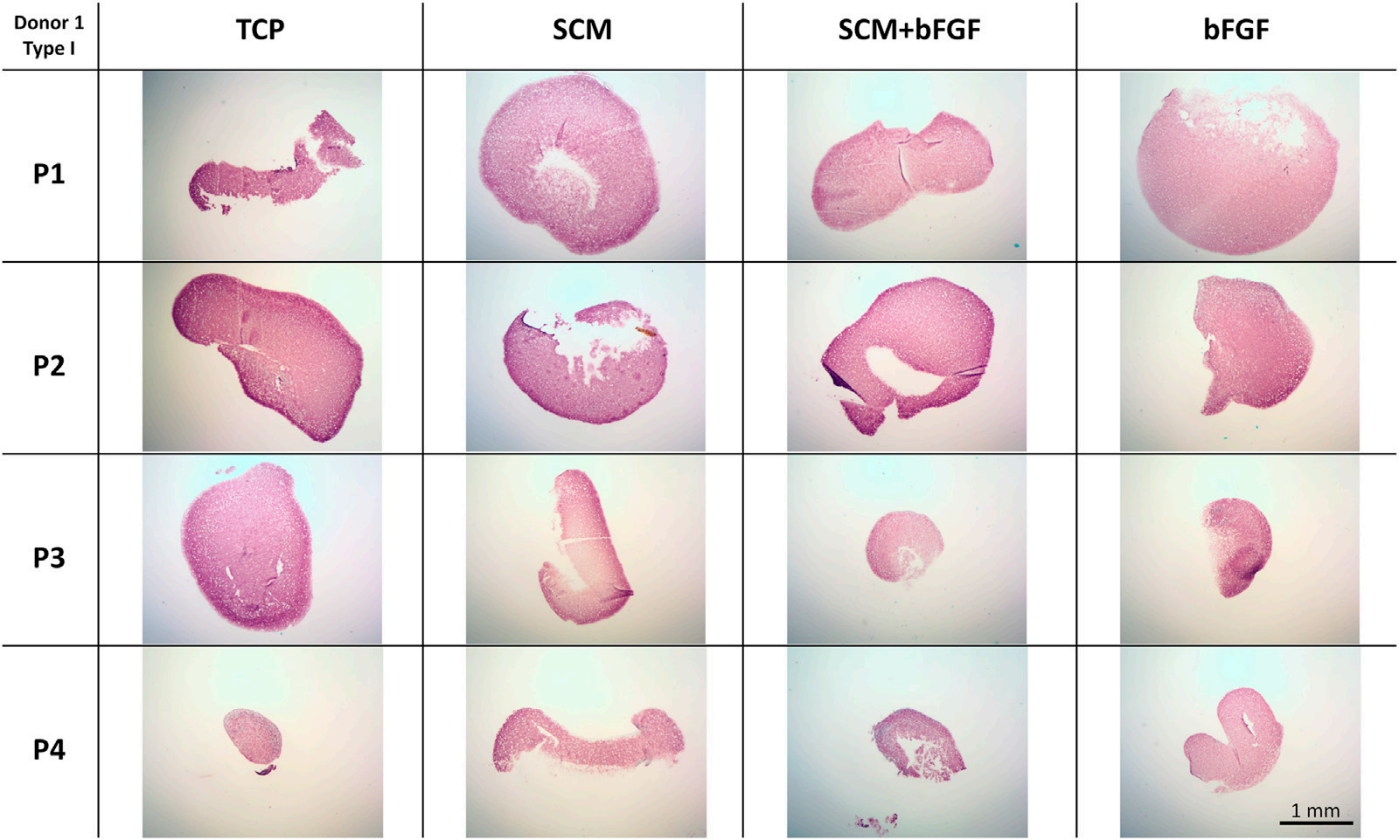
Cartilage aggregate images showing type I collagen staining. Sections of cartilage aggregates from a single donor over the four passages in each condition are shown here stained for type I collagen. All at the same scale, scale bar 1 mm. See Supplementary Figures S8–S10 for other donors.

Fibrous tissue formation, assessed by type I collagen staining, was also performed (Figure 9). Again, in all conditions tested, there was significant deposition of type I collagen. This was relatively stronger in the TCP group versus the other conditions. When looking across donors, there was a significant lack of staining in donor 5 (Supplementary Figure S10). In general, there was a trend to more intense staining as passages increased across all donors (Figure 9 and Supplementary Figures S8–S10).

**FIGURE 10:**
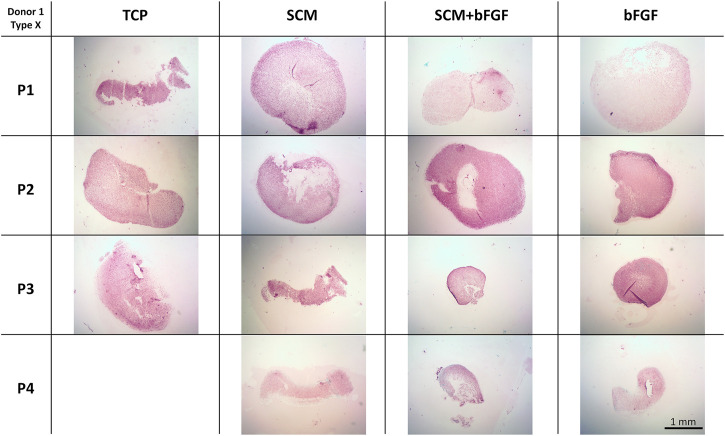
Cartilage aggregate images showing type X collagen staining. Sections of cartilage aggregates from a single donor over the four passages in each condition are shown here stained for type X collagen. All at the same scale, scale bar 1 mm. See Supplementary Figures S11–S13 for other donors.

Hypertrophic tissue formation, indicated by type X collagen, was also assessed (Figure 10). Type X collagen staining was more pronounced in TCP at P1 than in the other three conditions. The SCM had the least intense staining across all four passages. SCM + bFGF and bFGF had relatively strong staining at passage 2, with bFGF also having relatively strong staining at passage 3 (Figure 10). Staining in Donor 5 was quite weak overall with some stronger staining in TCP at P3 and P4; SCM at P1, P2, and P3; and FGF at P1 (Supplementary Figure S13). Across all donors, a general pattern of more intense staining in TCP was evident (Figure 10 and Supplementary Figures S11–S13).

## Discussion

In the field of human tissue engineering, bFGF has already been shown to lead to the expansion of human neural stem cells and has demonstrated an important role in the regulation of stem cell proliferation (Flax et al., 1998; Amit et al., 2000; Zhang et al., 2001). A previous study examined the effects of bFGF on the proliferation and phenotypic differentiation of human neural stem cells, both alone and in combination with the epidermal growth factor (EGF) and leukemia inhibitory factor (LIF), with results indicating that while bFGF did lead to increased cell proliferation, the combination of bFGF, EGF, and LIF led to a more efficient proliferation of human neural stem cells than any other growth environment (Tarasenko et al., 2004). bFGF has also been shown to lead to increased proliferation of human corneal cells, leading to accelerated wound healing. In particular, when added to the platelet-derived growth factor BB-isoform (PDGF-BB), the combination leads to faster wound closure than with bFGF or PDGF-BB individually (Gallego-Muñoz et al., 2017). Based on the aforementioned studies, bFGF in combination with other substances that promote tissue growth leads to an additive effect of cellular expansion than if bFGF was added alone to the growth environment, a notion that was reinforced by the findings of this study.

This study showed a significant improvement in human chondrocyte expansion and an increase in both GAG and collagen content in cartilage aggregates with all three test conditions compared with standard TC plates. The results show a significantly increased cell expansion rate between all three test conditions (SCM, SCM + bFGF, bFGF) and the control (TCP) (*p* < 0.0001), sustained throughout the four passages. This is consistent with our previous work that has shown enhanced growth of chondrocytes with the use of SCM compared to TCP (Kean et al., 2019; Dennis et al., 2020). In addition, chondrocytes cultured in SCM + bFGF showed a significantly higher expansion rate than in bFGF alone in all four passages, indicating that there is some additional benefit to including both components. The combination of SCM + bFGF resulted in approximately 36% more cells per passage (5–7 days) than in SCM alone. The SCM contains both the growth factors and provides an almost 3D surface for growth. The increased surface area along with additional growth factors could further stimulate proliferation more than that achieved with either SCM or bFGF alone. With the GAG and collagen content serving as positive correlative markers for cartilage production, these results imply an improved ability of chondrocytes to retain their differentiation potential, addressing a major obstacle in prior studies.

Another challenge encountered in tissue-engineered cartilage is that it is typically found to be deficient in type II collagen specifically, which provides tensile strength to the cartilage tissue and resistance against compression (Phull et al., 2016; Whitney et al., 2018). In our study, it was found that the growth environment containing SCM + bFGF had significantly greater collagen content in passages 3 and 4 than in all other conditions. This increase in collagen content was indicated in the hydroxyproline assay, which shows the amount of total collagen in the matrix. It is, therefore, possible that we could be observing an increase in the fibrous collagen content, specifically type I collagen, and a decrease in type II collagen. This was not evident in the immunohistochemistry data which had only minor correlations with the increased staining and passage. This increase in types I and X collagen and a decrease in type II were most apparent in the TCP group. In a previous work, it was observed that the type I collagen in our tissue-engineered cartilage reduced with increased culture time to 2 months, something we did not investigate here (Dennis et al., 2020).

It should also be noted that the GAG concentration increased over passages 1 to 3 in all conditions, which is contrary to most present literature (Schmal et al., 2007). For this phenomenon, we have three possible explanations: improved isolation of the chondrocyte tissue, improved cryopreservation and recovery of the tissue, and culturing in physiologic oxygen tension from day 0. In this study, we collected and incubated the tissue at room temperature in defined chondrogenic media, rather than in PBS on ice (Cook et al., 2014). In addition, we chose to sequentially digest the tissue first in hyaluronidase and then collagenase rather than hyaluronidase, trypsin, and collagenase as we had found trypsin/EDTA to have negative effects on the cell yield (data not shown). We also found that we could preserve the cells just as well, if not better, in 5% DMSO versus 10% (data not shown). Finally, as was shown in several previous studies, culturing the cells and tissues in physiologic oxygen tension led to increased extracellular matrix deposition and increased mechanical properties of the tissue-engineered cartilage. The cells and tissues were, therefore, cultured in physioxia from day 0 of the study which could have greatly contributed to an advantageous environment for chondrocyte cell growth. This seems to be particular to the articular cartilage as we found a negative effect of 5% oxygen in auricular chondrocytes (Kean et al., 2016; Dennis et al., 2018). All of the aforementioned minor modifications have previously been shown to be potentially advantageous for chondrocyte culture growth, and considering the results of this study, they certainly seem to be of benefit.

As previously mentioned, preserving the ability of the chondrocytes to differentiate is one of the greatest challenges of cartilage tissue engineering. Treatment of OA lesions has proven to be challenging, considering that ACI and MACI have both been developed as excellent treatment strategies that do not require total joint replacement surgery but that tissue created from these methods often fails to maintain the differentiation ability of chondrocytes producing fibrous tissue (Giovannini et al., 2010). This loss of differentiation ability leads to an eventual decline in hyaline cartilage production, thus leading to a delay of, rather than the prevention of, total joint replacement surgery. However, this study has shown that sustained chondrocyte growth is, indeed, possible through the use of SCM + bFGF growth media. It, therefore, supports the idea that such an environment is conducive and advantageous for maintaining chondrocyte differentiation ability, marking an important advancement in the field of joint regeneration. Although adding bFGF to the healing process may raise safety concerns, several studies have shown that the addition of growth factors, including bFGF, IFG-I, and TGF-β1, to the treatment of bone fractures enhanced the healing process while having an excellent safety profile (Schmidmaier et al., 2004; Kawaguchi et al., 2007).

Several previous studies have examined the effects of other FGFs, including FGF-18 and a sequence of FGF-2, 9, and 18 on the growth and differentiation of MSCs and chondrocytes. Shimoaka et al. (2002) found that compared with FGF-2, FGF-18 had similar stimulatory action on osteoclast bone resorption and mitogenic activation of osteoblasts and chondrocytes as FGF-2, indicating that FGF-18 could compensate for the roles FGF-2 plays in normal physiology. Future directions of this study could include the use of other FGFs, such as FGF-18, in combination with the SCM to examine if another FGF could potentially outperform bFGF in terms of enhancing chondrocyte proliferation potential. The results of this study can eventually lead to major improvements in the lifespan of ACI and MACI, thus allowing for a decrease in the need for eventual total joint replacement. Larger or even total joint resurfacing *via* ACI, MACI, or 3D bioprinting could also become distinct possibilities. Other directions of the study include the potential for the creation of a larger cell reservoir to treat multiple cartilage tissue lesions from a single, potentially smaller, biopsy and possible pre-implantation testing for chondrogenicity.

## Conclusion

Human articular chondrocyte tissues cultured in environments enhanced with bFGF, SCM, and a combination of the two have shown significant improvement in expansion potential when compared to tissue cultured on typical culture plastic alone. In particular, bFGF + SCM was shown to perform the best overall, with 36% more cells per passage than with the SCM alone. An observed increase in the GAG and collagen content in all the growth mediums is also indicative of the improved ability of cultured chondrocytes to maintain their differentiation ability and therefore marks an important step forward in the field of tissue engineering.

## Data Availability

The original contributions presented in the study are included in the article/Supplementary Material, further inquiries can be directed to the corresponding author.

## Supplementary Material

Supplementary Figure S1 Correlation of the decreased aggregate wet weight with increased population doublings. There was a negative correlation of the aggregate wet weight with increased population doublings **(A)** TCP, **(B)** SCM, **(C)** SCM+bFGF, and **(D)** bFGF.

Supplementary Figure S2 Cartilage aggregate images of donor 2 showing glycosaminoglycan (GAG) staining. The sections of cartilage aggregates from donor 2 over the four passages on each surface are shown here stained with safranin-O/Fast Green. All at the same scale, scale bar 1 mm. See Supplementary Figures S2–S4 for other donors.

Supplementary Figure S3 Cartilage aggregate images of donor 3 showing glycosaminoglycan (GAG) staining. Sections of cartilage aggregates from donor 3 over the four passages on each surface are shown here stained with safranin-O/Fast Green. All at the same scale, scale bar 1 mm. See Supplementary Figures S2–S4 for other donors.

Supplementary Figure S4 Cartilage aggregate images of donor 5 showing glycosaminoglycan (GAG) staining. Sections of cartilage aggregates from donor 5 over the four passages on each surface are shown here stained with safranin-O/Fast Green. All at the same scale, scale bar 1 mm. See Supplementary Figures S2–S4 for other donors.

Supplementary Figure S5 Cartilage aggregate images of donor 2 showing type II collagen staining. Sections of cartilage aggregates from donor 2 over the four passages in each condition are shown here stained for type II collagen. All at the same scale, scale bar 1 mm. See Supplementary Figures S5–S7 for other donors.

Supplementary Figure S6 Cartilage aggregate images of donor 3 showing type II collagen staining. Sections of cartilage aggregates from donor 3 over the four passages in each condition are shown here stained for type II collagen. All at the same scale, scale bar 1 mm. See Supplementary Figures S5–S7 for other donors.

Supplementary Figure S7 Cartilage aggregate images of donor 5 showing type II collagen staining. Sections of cartilage aggregates from donor 5 over the four passages in each condition are shown here stained for type II collagen. All at the same scale, scale bar 1 mm. See Supplementary Figures S5–S7 for other donors.

Supplementary Figure S8 Cartilage aggregate images of donor 2 showing type I collagen staining. Sections of cartilage aggregates from donor 2 over the four passages in each condition are shown here stained for type I collagen. All at the same scale, scale bar 1 mm. See Supplementary Figures S8–S10 for other donors.

Supplementary Figure S9 Cartilage aggregate images of donor 3 showing type I collagen staining. Sections of cartilage aggregates from donor 3 over the four passages in each condition are shown here stained for type I collagen. All at the same scale, scale bar 1 mm. See Supplementary Figures S8–S10 for other donors.

Supplementary Figure S10 Cartilage aggregate images of donor 5 showing type I collagen staining. Sections of cartilage aggregates from donor 5 over the four passages in each condition are shown here stained for type I collagen. All at the same scale, scale bar 1 mm. See Supplementary Figures S8–S10 for other donors.

Supplementary Figure S11 Cartilage aggregate images of donor 2 showing type X collagen staining. Sections of cartilage aggregates from donor 2 over the four passages in each condition are shown here stained for type X collagen. All at the same scale, scale bar 1 mm. See Supplementary Figures S11–S13 for other donors.

Supplementary Figure S12 Cartilage aggregate images of donor 3 showing type X collagen staining. Sections of cartilage aggregates from donor 3 over the four passages in each condition are shown here stained for type X collagen. All at the same scale, scale bar 1 mm. See Supplementary Figures S11–S13 for other donors.

Supplementary Figure S13 Cartilage aggregate images of donor 5 showing type X collagen staining. Sections of cartilage aggregates from donor 5 over the four passages in each condition are shown here stained for type X collagen. All at the same scale, scale bar 1 mm. See Supplementary Figures S11–S13 for other donors.
