# Supplementary figures and images for: Synoviocyte-Derived Extracellular Matrix and bFGF Speed Human Chondrocyte Proliferation While Maintaining Differentiation Potential
Source: Front Bioeng Biotechnol. 2022 May 24;10:825005. doi: 10.3389/fbioe.2022.825005 (PMC9171110; doi:10.3389/fbioe.2022.825005)

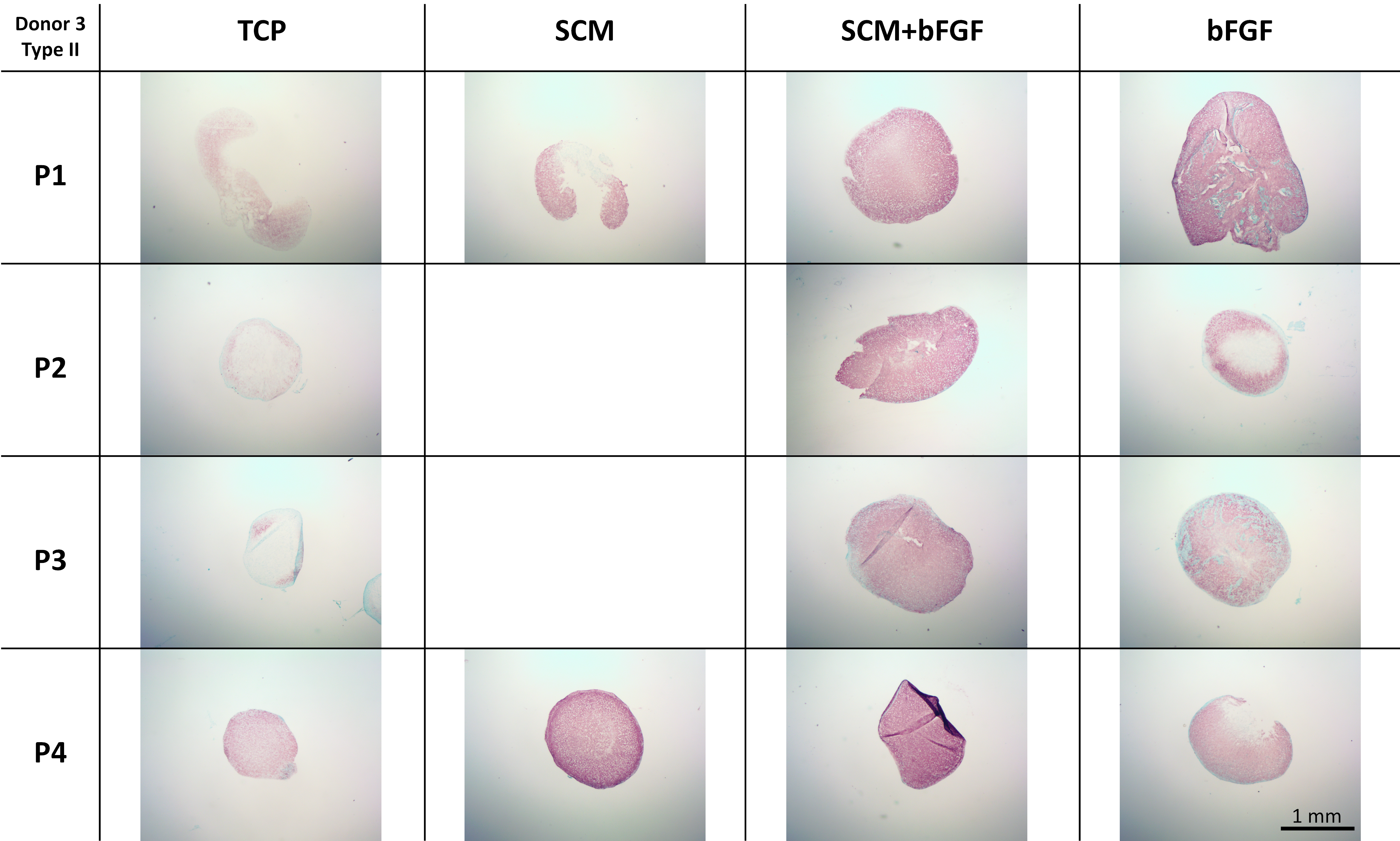

Supplement: Supplementary file 1 [file Image6.TIF]

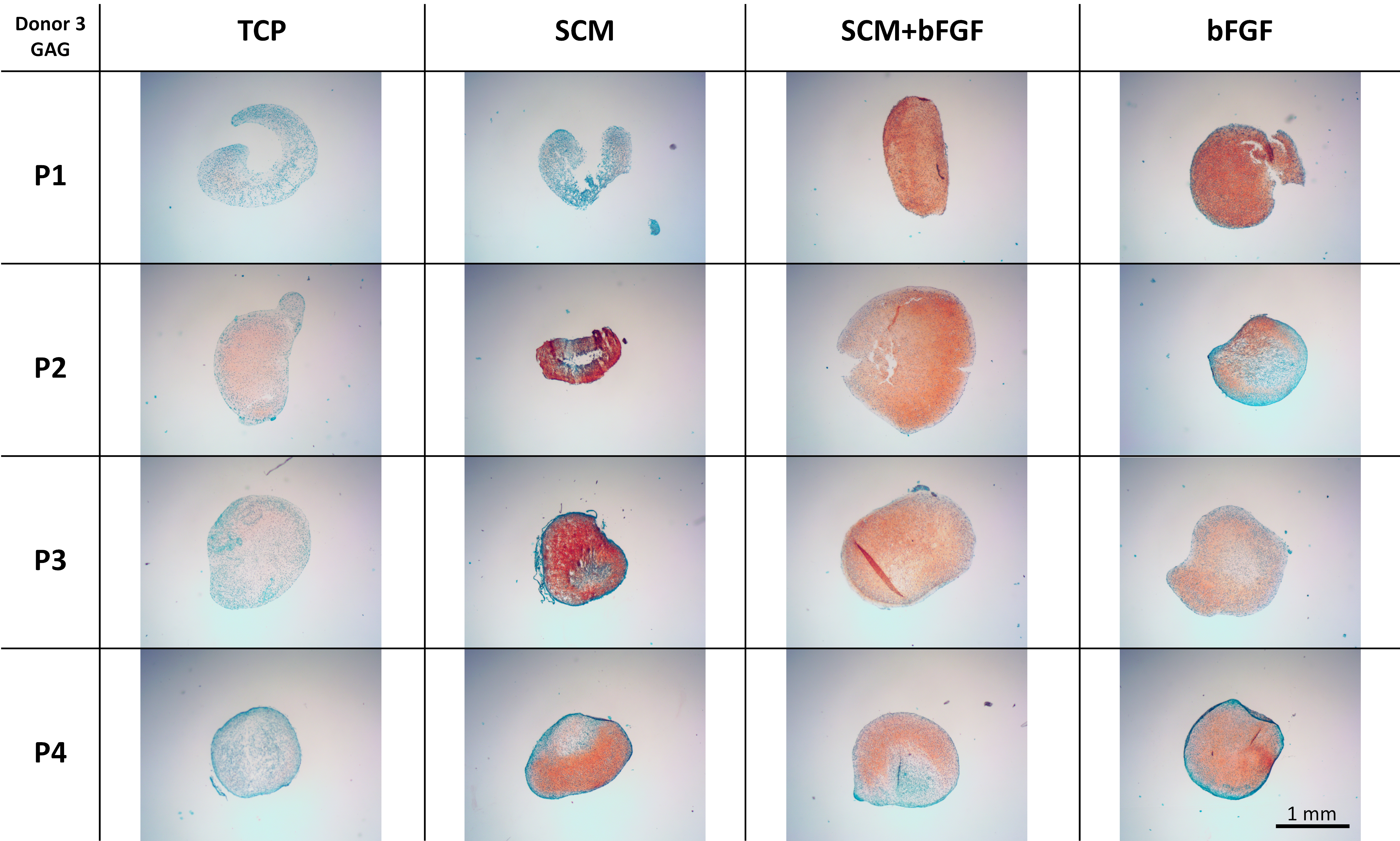

Supplement: Supplementary file 2 [file Image3.TIF]

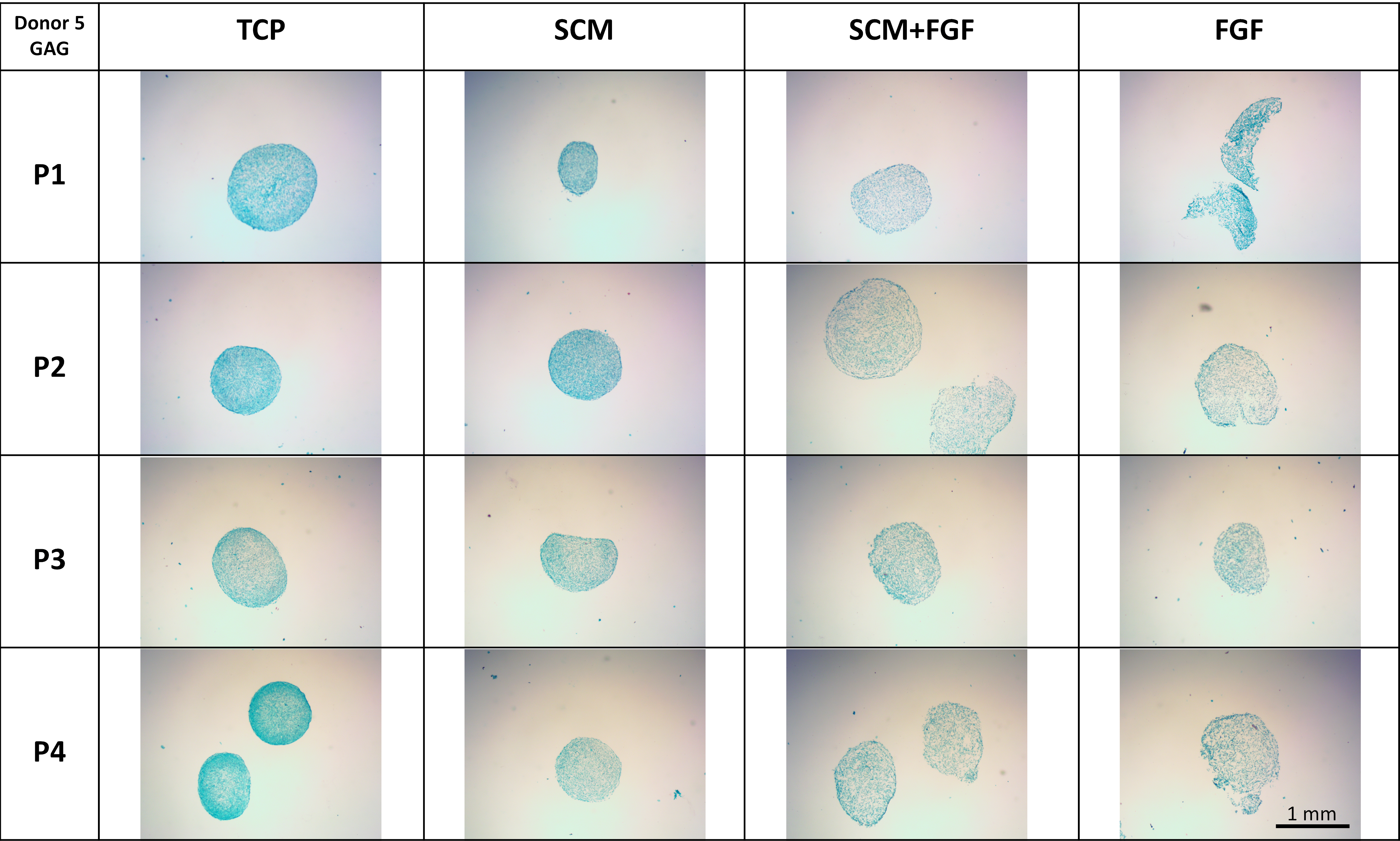

Supplement: Supplementary file 3 [file Image4.TIF]

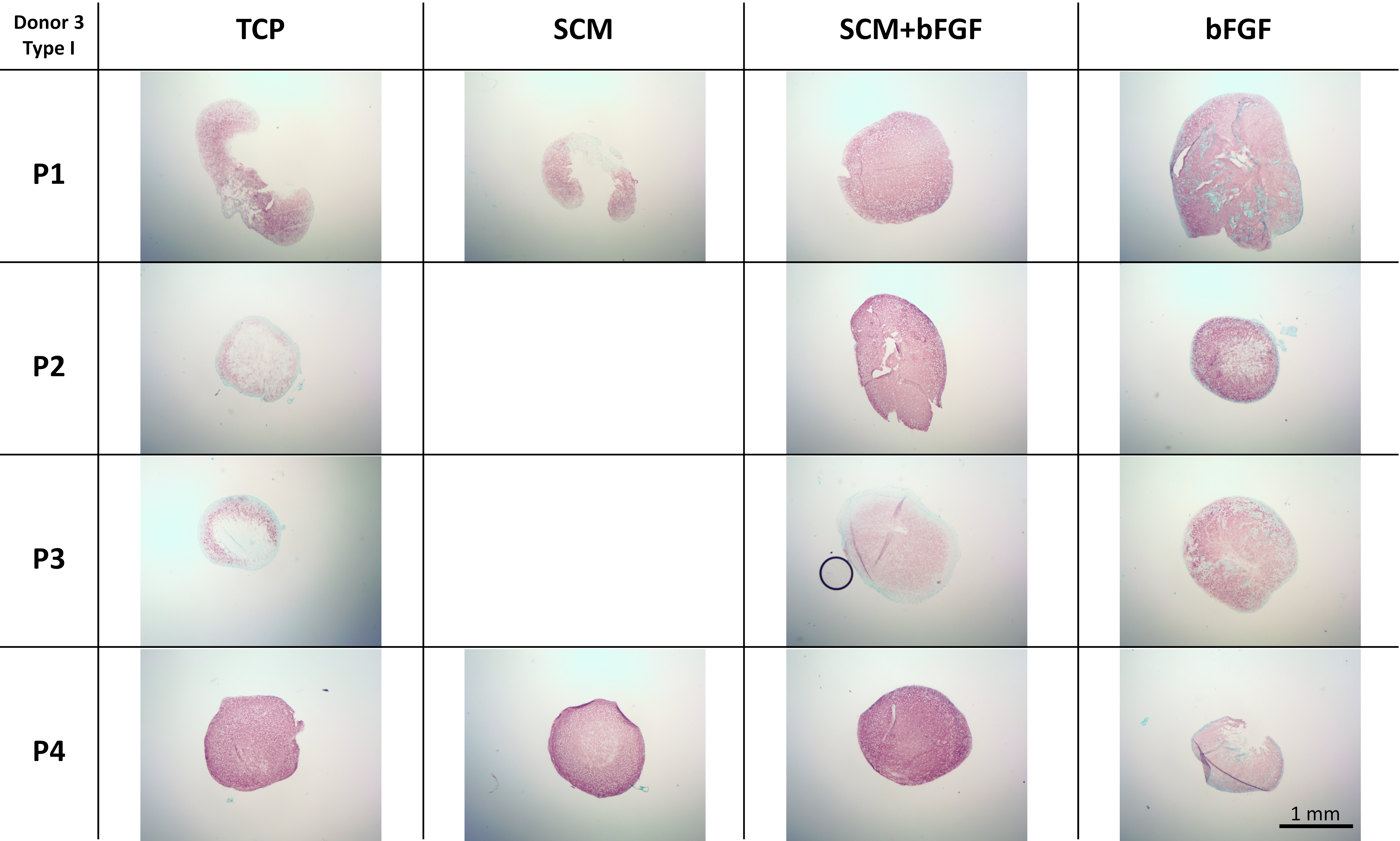

Supplement: Supplementary file 4 [file Image9.TIF]

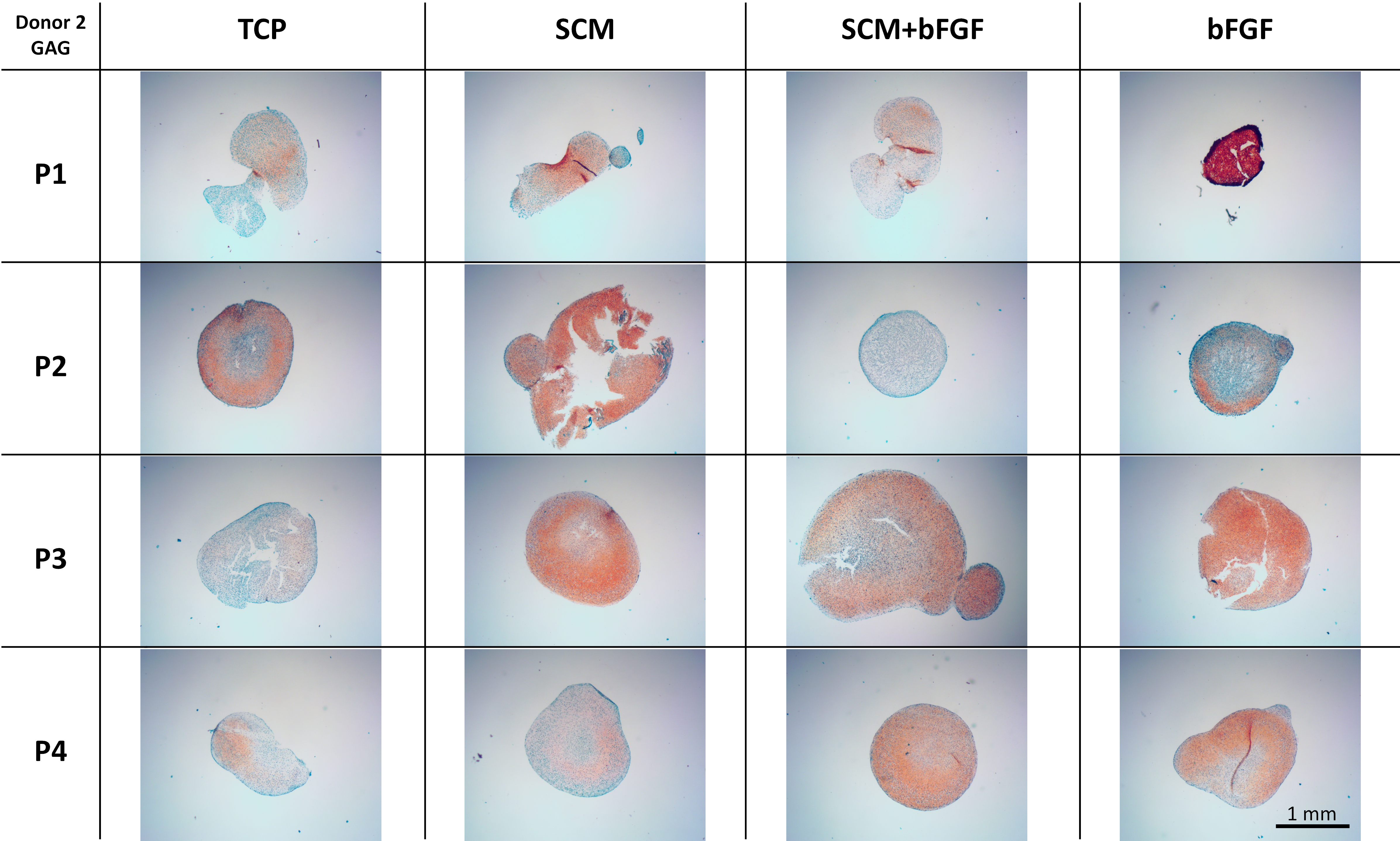

Supplement: Supplementary file 5 [file Image2.TIF]

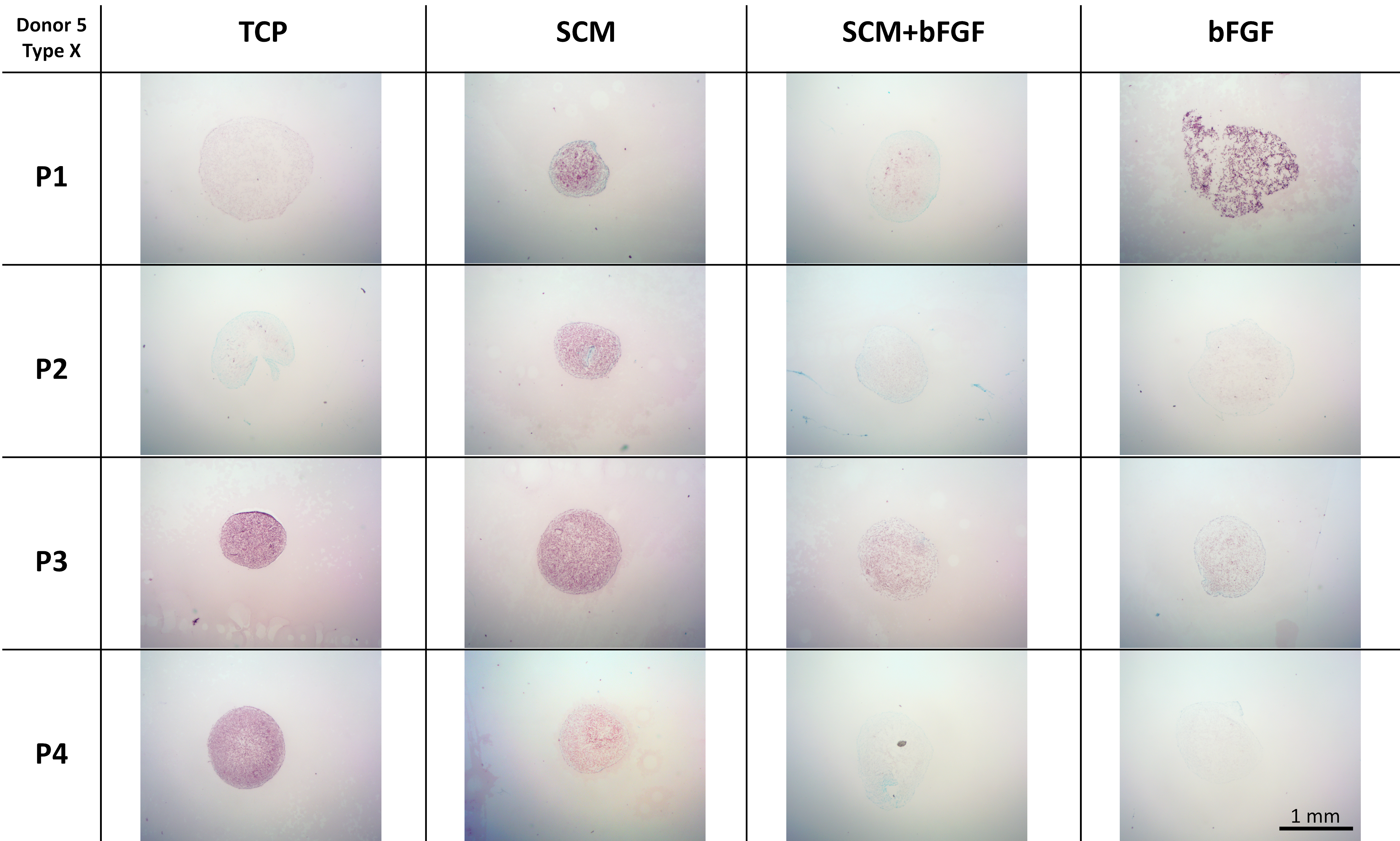

Supplement: Supplementary file 6 [file Image13.TIF]

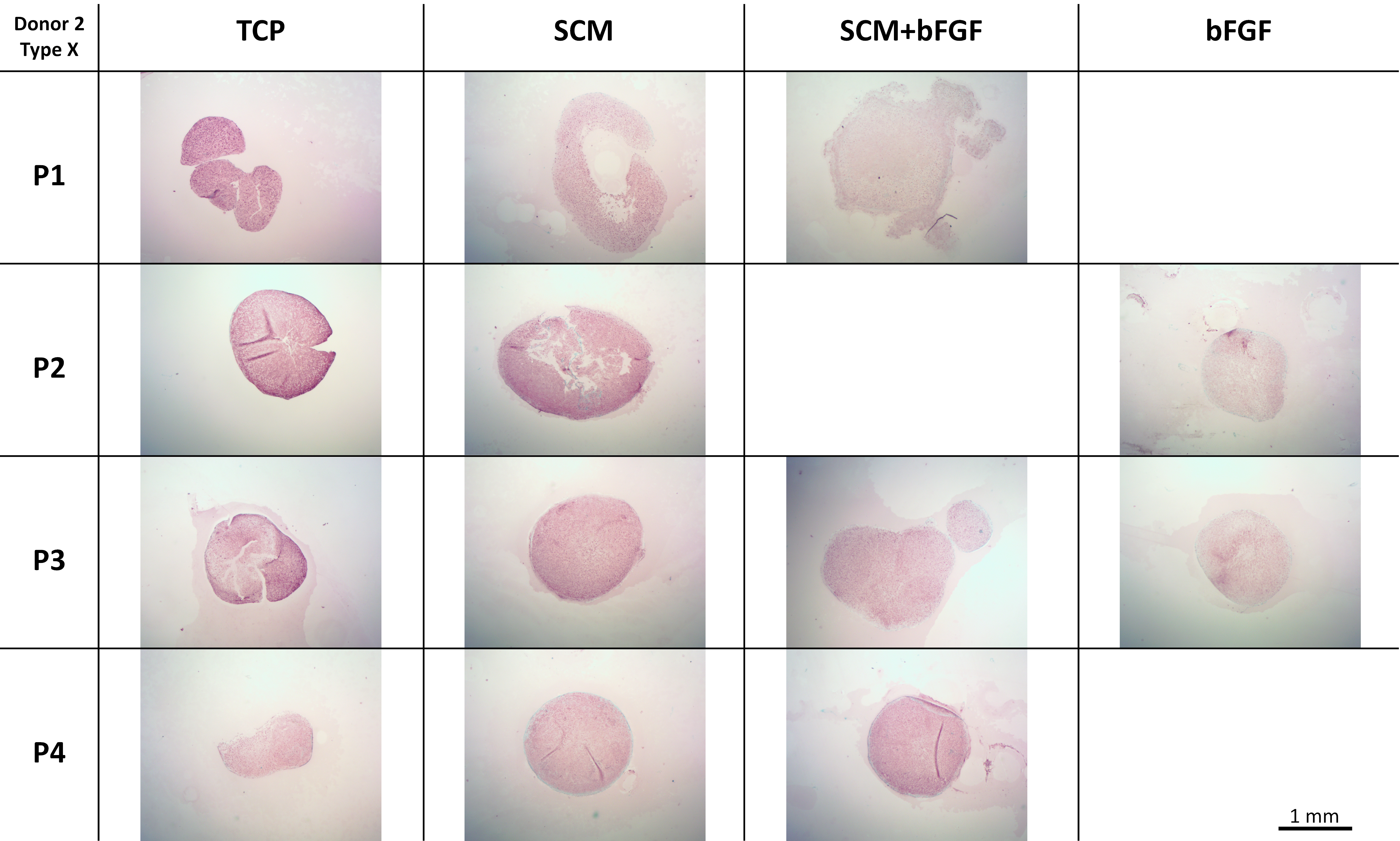

Supplement: Supplementary file 7 [file Image11.TIF]

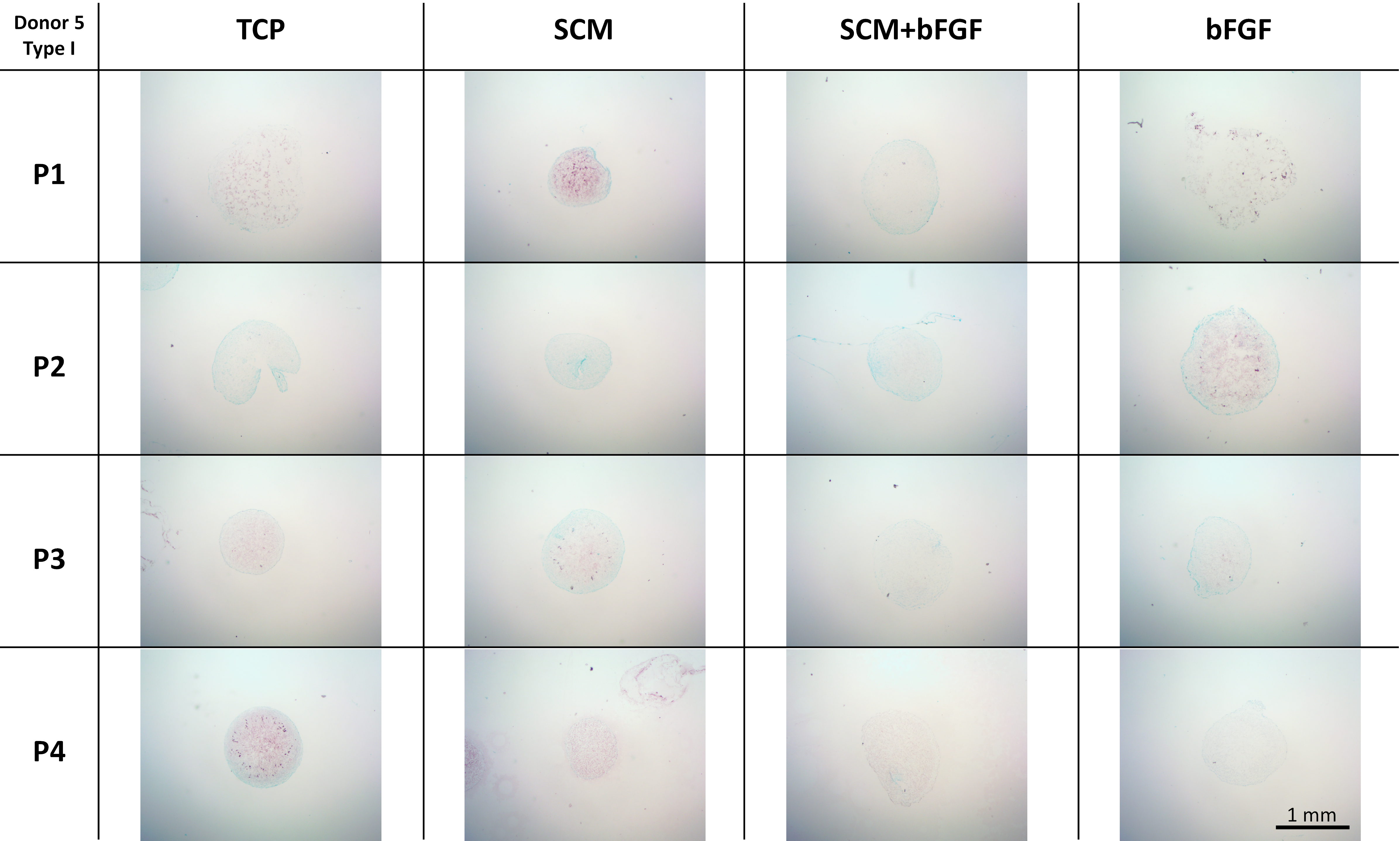

Supplement: Supplementary file 9 [file Image10.TIF]

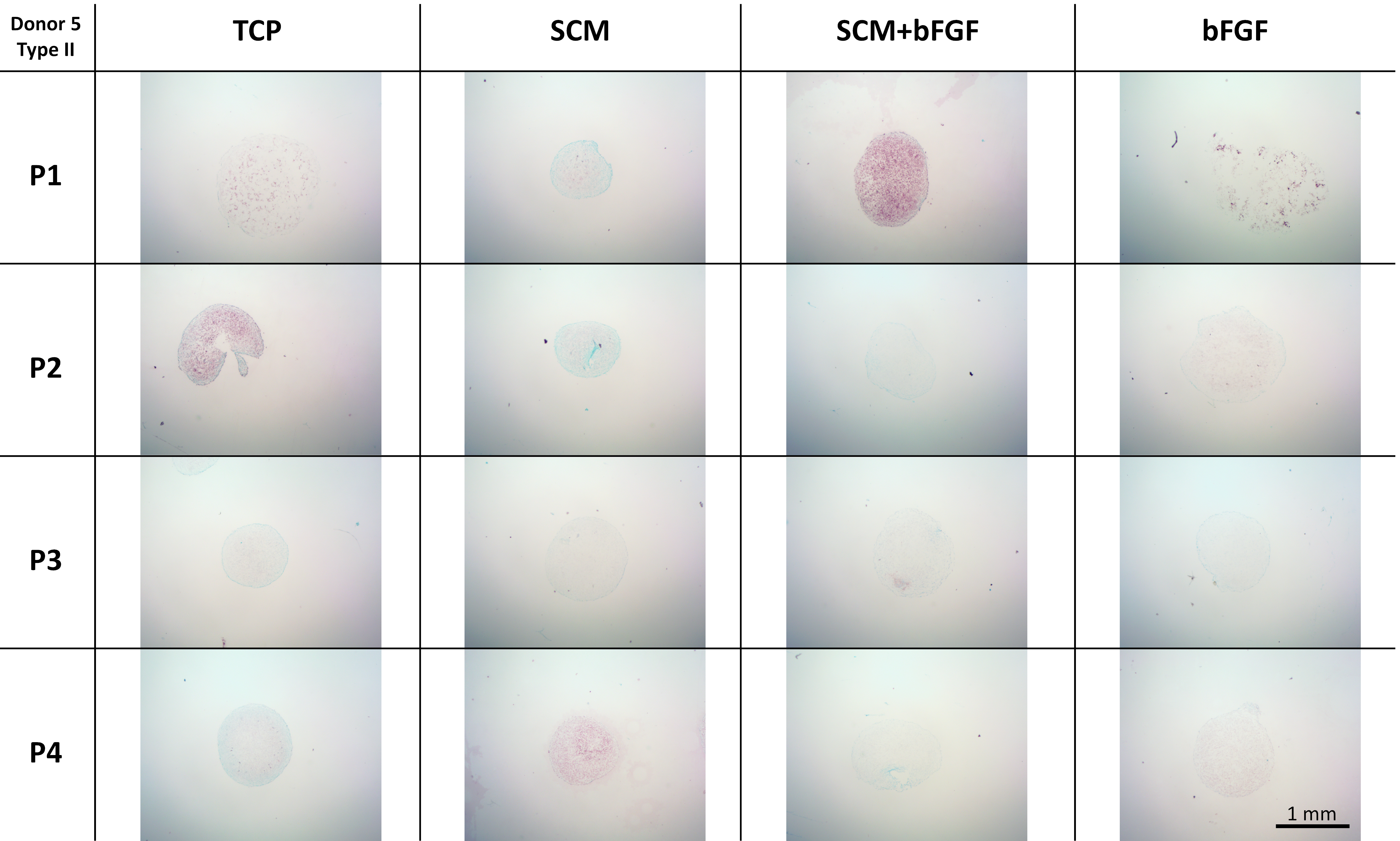

Supplement: Supplementary file 10 [file Image7.TIF]

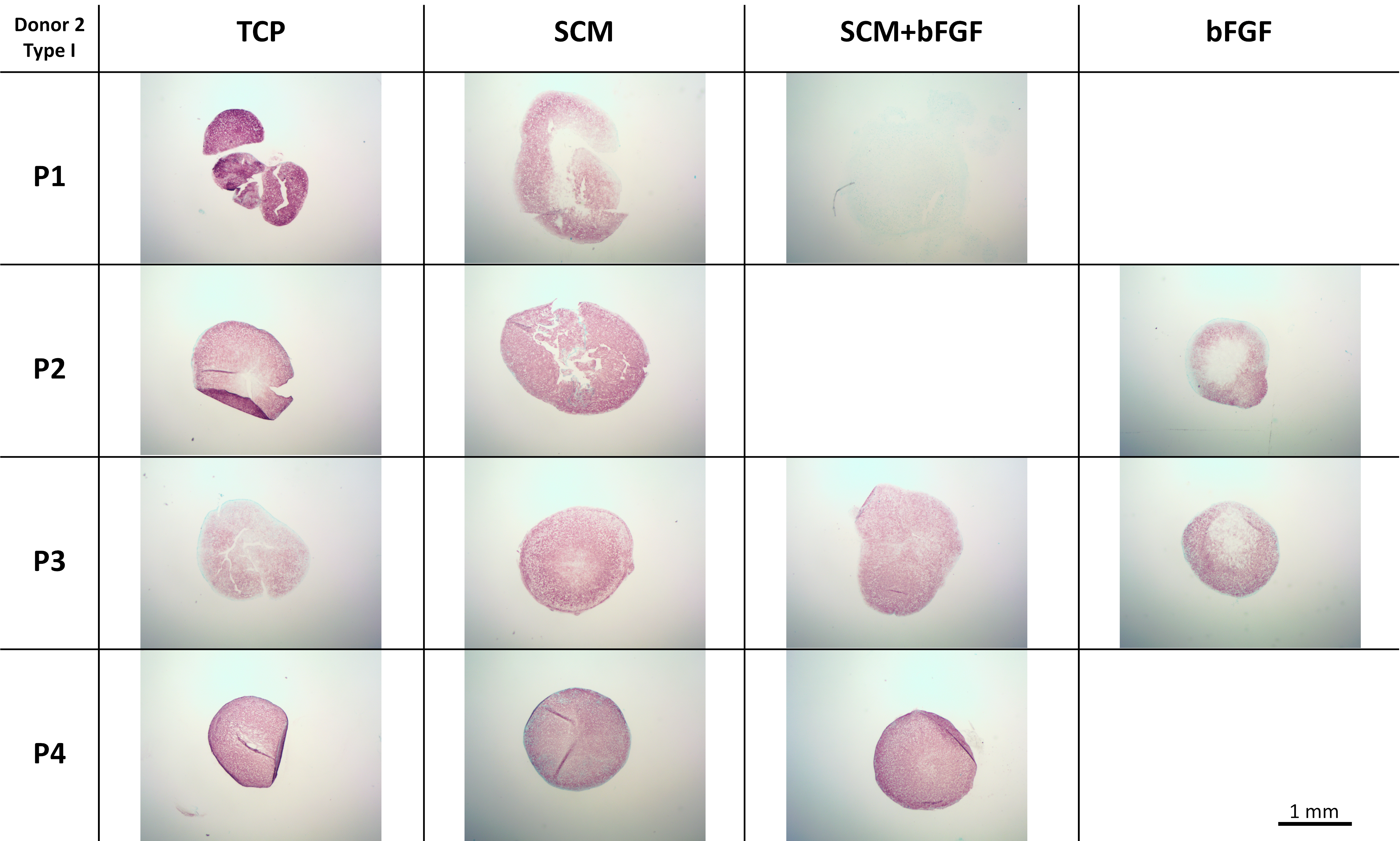

Supplement: Supplementary file 11 [file Image8.TIF]

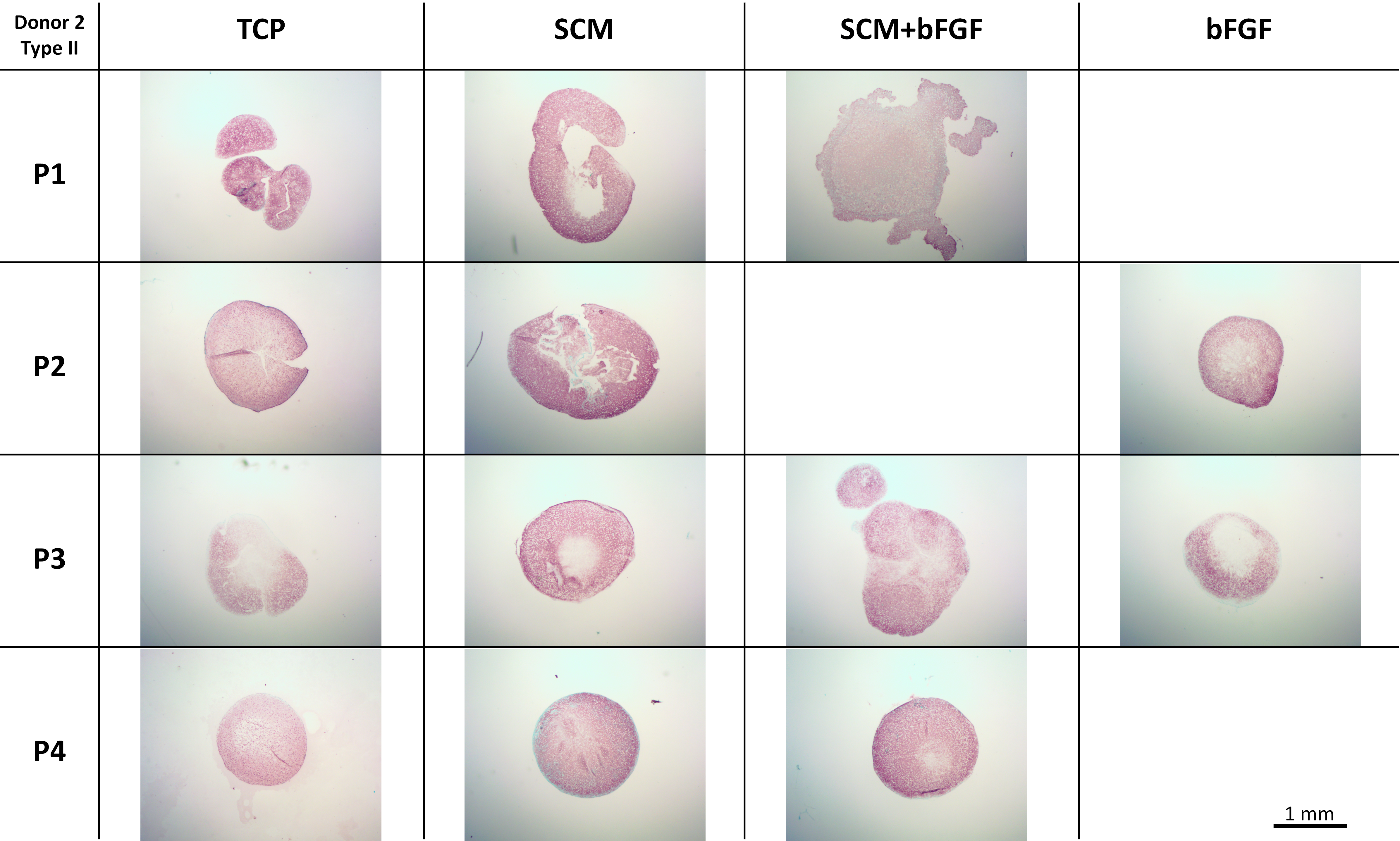

Supplement: Supplementary file 12 [file Image5.TIF]

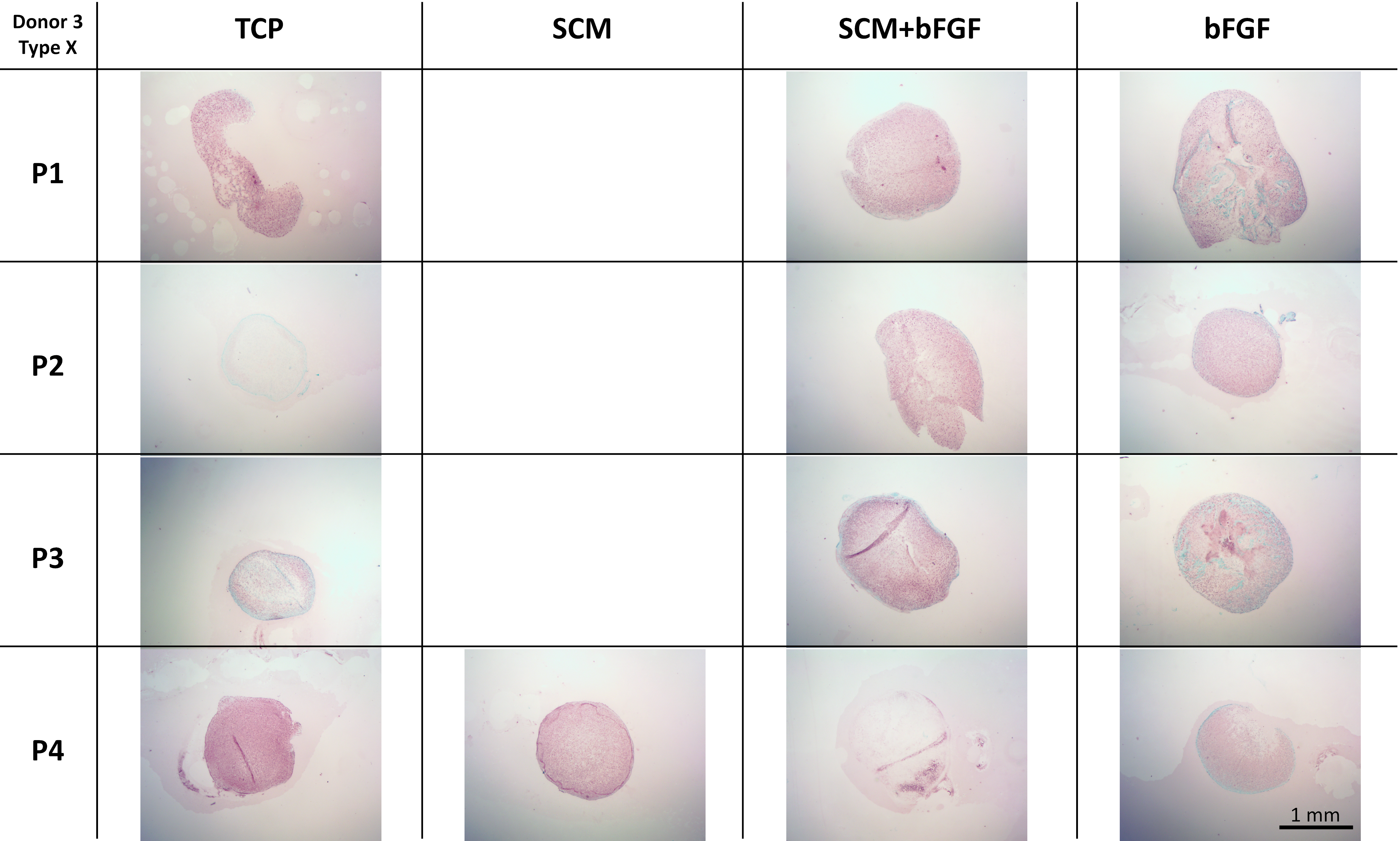

Supplement: Supplementary file 13 [file Image12.TIF]
